# Development of Collaterals in Intermittent and Permanent Ischemia of the Liver

**DOI:** 10.1155/1996/94108

**Published:** 1996

**Authors:** Engin Ok, Zeki Yilmaz, Erhan Akgün, Erdoğan M. Sözüer, Yaşar Yeşilkaya, Figen Öztürk

**Affiliations:** Departments of Surgery and Pathology University of Erciyes Kayseri Turkey

## Abstract

The ischemia caused by hepatic dearterialization as therapy for hepatic malignancies is transient because of the rapid formation of collaterals. In order to prevent this transient repeated ischemia has been suggested.

An experimental study was planned to compare the collateral occurrence in persistent ischemia and transient repeated ischeamia of the liver. Fourteen dogs (seven persistent ischemia, seven transient repeated ischemia) were used in this study. Hepatic dearterialization were performed in both groups. In the first group (persistent ischemia), the hepatic artery was ligated proximal to the gastroduodenal artery. In the second group (transient repeated ischemia), the hepatic artery was occluded externally in the same region as the first group by means of a device modified from 8 guage Foley catheter and after occlusion for one hour it was reopened. Occlusions were repeated twice in a day. Five dogs in the first group and six dogs in the second group completed a three weeks ischemia period and angiography were then performed in all. The dogs were sacrificed after the angiography and examined for possible abscess formation, arterial thrombosis, peritoneal adhesions and liver necrosis. After angiography, the two groups were also examined for collateral occurrence. Only one collateral occurred in the transient repeated ischemia group, but in the persistent ischemia group, collaterals occurred in all dogs. This difference between two groups is statistically significant (Fischer Absolute Chi Square Test, p=0.013).

Transient repeated ischemia is superior to persistent ischemia because of fewer collaterals, but in practise, total dearterialization of the liver is impossible.


*HPB Surgery*, 1996, Vol.10, pp.35–40
Reprints available directly from the publisher
Photocopying permitted by license only

© 1996 OPA (Overseas Publishers Association)
Amsterdam B.V. Published in The Netherlands
by Harwood Academic Publishers GMbH
Printed in Malaysia

### Development of Collaterals in Intermittent and Permanent Ischemia of the Liver*

ENGIN OK, ZEKI YILMAZ, ERHAN AKGÜN, ERDOĞAN M. SÖZÜER, YAŞAR YEŞILKAYA and FIGEN ÖZTÜRK[1]

Departments of Surgery and Pathology[1], University of Erciyes, Kayseri, Turkey

*(Received 23 October 1993)*

The ischemia caused by hepatic dearterialization as therapy for hepatic malignancies is transient because of the rapid formation of collaterals. In order to prevent this transient repeated ischemia has been suggested.

An experimental study was planned to compare the collateral occurrence in persistent ischemia and transient repeated ischeamia of the liver. Fourteen dogs (seven persistent ischemia, seven transient repeated ischemia) were used in this study. Hepatic dearterialization were performed in both groups. In the first group (persistent ischemia), the hepatic artery was ligated proximal to the gastroduodenal artery. In the second group (transient repeated ischemia), the hepatic artery was occluded externally in the same region as the first group by means of a device modified from 8 guage Foley catheter and after occlusion for one hour it was reopened. Occlusions were repeated twice in a day. Five dogs in the first group and six dogs in the second group completed a three weeks ischemia period and angiography were then performed in all. The dogs were sacrificed after the angiography and examined for possible abscess formation, arterial thrombosis, peritoneal adhesions and liver necrosis. After angiography, the two groups were also examined for collateral occurrence. Only one collateral occurred in the transient repeated ischemia group, but in the persistent ischemia group, collaterals occurred in all dogs. This difference between two groups is statistically significant (Fischer Absolute Chi Square Test, p=0.013).

Transient repeated ischemia is superior to persistent ischemia because of fewer collaterals, but in practise, total dearterialization of the liver is impossible.

KEY WORDS: Ischemia liver intermittent ischemia

### INTRODUCTION

Use of ischemia to produce necrosis is a relatively new approach in the treatment of hepatic tumors. The objective in this appraoch is to protect the normal hepatic parenchymal structures while maximizing the damage to tumor cells. Preservation of the normal parenchyme is attributed to the dual blood supply of the liver. Meanwhile, maximum damage to tumor cells is dependant on the fact that the major supply of liver tumors is by arteries[1,2,5]. However, permanent obstruction of the hepatic artery results in swift development of collateral branches; thus preventing effective and successful results[12,20,24,30,31,44]. After the importance of oxygen-derived free radicals was clarified in relation to the physiopathology of ischemia-reperfusion, it was found that intermittent ischemia proved to be much more effective than permanent ischemia[42,46]. The dual blood flow to the liver explains the swift formation of oxygen-derived free radicals. When the hepatic artery is ligated, the partial oxygen pressure in the portal vein is sufficient to provide both ischemic changes and development of oxygen-derived free radicals[42]. Supported by the studies carried out by Mack *et al.*, this view has put forward the advantages and superiority of intermittent ischemia attacks over permanent ischemia in treatment of hepatic tumors[27].

*This Study was presented in 1st UNITED EUROPEAN GASTROENTEROLOGY WEEK, ATHENS, September 25–30, 1992 as an oral presentation.

*Correspondence to*: Dr Engin Ok, Department of Surgery, SSK Hospital, Kayseri, Türkiye

### MATERIALS AND METHODS

In this study 14 dogs, 7 male and 7 female, and weigh in between 11 and 28 kg (average: 20.5 kg) were used. Two groups were formed, each including 7 dogs. One dog was used for control. Subjects were left overnight without food or water.They were anesthetized with 25 mg/kg thiopentone sodium and given an intravenous injection of 100 mg/kg third generation cepholosporin.

Under a an aesthesial abdomen was opened through a mid-line incision. All connections; falciform, coronary and triangular ligaments, the lesser omentum, portal vein, common bile duct and hepatic artery within the hepatoduodenal ligament, were desserted out. The hepatic artery was isolated upto the point where it entered the liver. Full mobilization of the liver was achieved through sharp and blunt dissection until the hepatic veins were reached. Through this method the liver was freed of all arterial connections and dearterialization was accomplished. In order to avoid necrosis, the gallbladder was removed in all subjects.

In the first group (permanent ischemia group), the hepatic artery was ligated proximal to the gastroduodenal artery using No. 1 Vicryl. In the second group, in order to attain intermittent ischemia, a device prepared with a No. 8 Foley catheter was placed proximal to the gastroduodenal artery (Fig. 1). The cuff of the device was wrapped around the hepatic artery and was fixed with fine non-absorbable sutures. The balloon of the Foley catheter was inflated and by external pressure arterial flow was completely obstructed. The amount offluid sufficient to stop theflow was recorded. The gastroduodenal artery was catheterized by a 0.8 × 1.4 mm, 45 cm radio-opaque silicon catheter. After bleeding was checked, the tips of the catheter and Foley catheter were laben to the surface of the abdomen, after which they were fixed to the abdominal skin. In order to prevent any possible damage, the catheters were placed in previously prepared metallic covers. After 24 hours, only water was given orally to the dogs; and on the second day, their normal diet was begun. In the second group, beginning from the forth day after the operation, the hepatic artery was obstructed twice a day for a period of one hour. During these periods no sedatives were given to the subjects. Antibiotics were administered until the 7th postoperative day.

At the end of the third week, angiographs were taken. Subjects were anesthetized with pentothal before angiography, and Urografin %76 ™ was manually injected by a 10 ml injector into the heparinized catheter which had been placed beforehand. Under screening, angiographs were taken at the time when dispersion was optimal.

At post-mortem exam in action a check was made for abscess or adhesion, and thrombus within the hepatic artery, after which liver biopsies were taken.

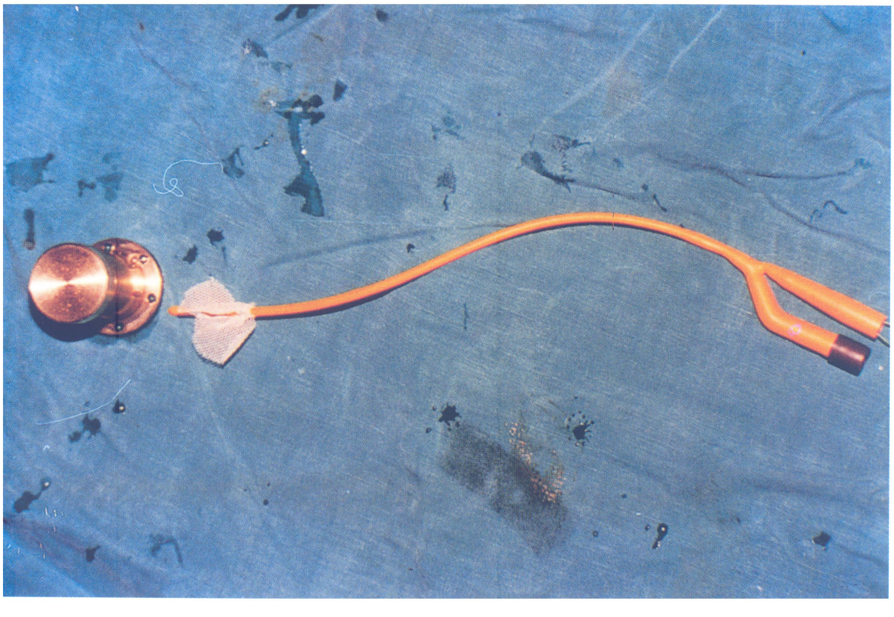

**Figure 1** To achieve intermittent ischemia, a special device was prepared by using a no: 8 Foley catheter (See color plate I)

The encouraging findings from our study, supported by data from the literature, led us to use intermittent ischemia to the liver in the treatment of a patient with a retroperitoneal malignant mesenchymal tumor with liver metastases, and in addition a pathological fracture of the humerus. The intermittent ischemia was performed after the tumor had been removed. The device which was used to occlude the hepatic artery by means of external pressure (Vascular occluder, Fig. 2) was obtained from Nelato Medical Co., Torekov, Sweden. Occlusions were initiated on the 12th day following the operation, together with chemotherapy treatment which was in accordance with the CyVADIC protocol.

### RESULTS

In the first group (permanent ischemia) two subjects died two days after the operation. In the second group (intermittent ischemia) one subject died, again at the second day following the operation.

The most frequent complication in the intermittent ischemia group was infection around the catheter covering. It was found during angiography that the catheters in two subjects, one from each group, had from the artery and were situated within the abdomen. These subjects were reoperated and the catheters replaced for immediate angiography.

Six subjects in the intermittent ischemia group, completed a period of three weeks treatment underwent angiography, collateral development was seen in only one subject (Fig. 3).

In the permanent ischemia group, angiographs were taken from 5 subjects which completed the three week period, and it was found that all 5 subjects presented recent arterial development together with collateral vessels.

In considering collateral development, a significant statistical distinction between the permanent and intermittent ischemia groups was noted, by use of Fisher's Chi Square Test ($p = 0.013$) (Table.1).

One subject from the intermittent ischemia group developed a subhepatic (in the gallbladder bed) and a subdiaphragmatic abscess. This subject also had intraabdominal adhesions. Adhesions were minimal in

**Table 1** Collateral development

| *Group* | *Collateral* | | *Total* |
|---|---|---|---|
| | *[+]* | *[-]* | |
| II | 1<br>(20%) | 5<br>(80%) | 6<br>(100%) |
| PI | 5<br>(100%) | 0<br>(0%) | 5<br>(100%) |
| TOTAL | 6<br>(54.5%) | 5<br>(45.5%) | 11<br>(100%) |

II = Intermittent ischemia
PI = Permanent ischemia

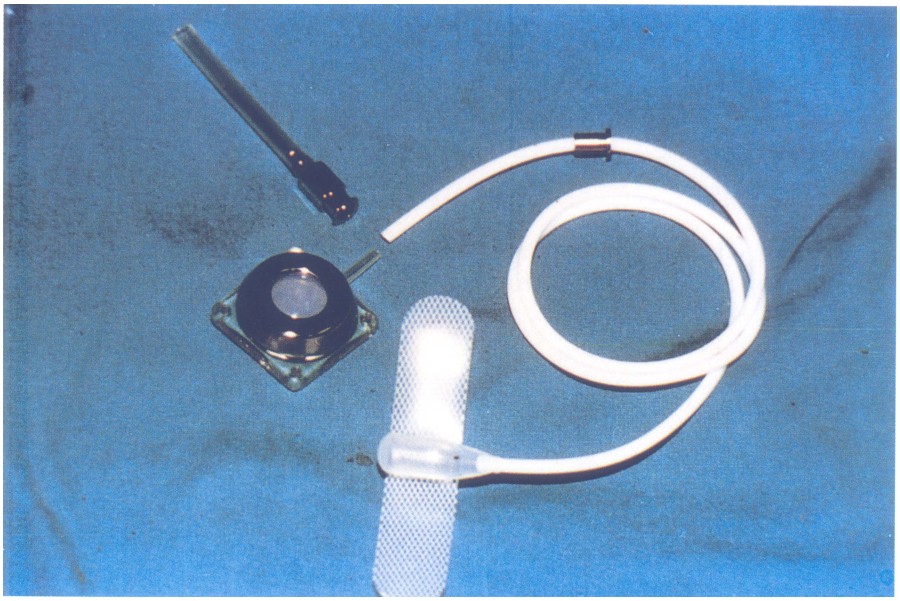

**Figure 2** Vascular occluder assured from Nolato Medical Co., Torekov, Sweden. (See color plate II)

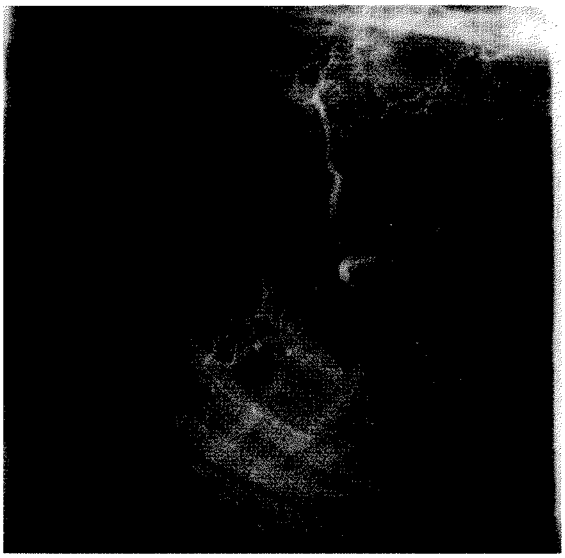

**Figure 3** Samples from intermittent ischemia group dogs, which there isn't any collateral formation.

other subjects of this group. Additionally, biopsies taken from the intermittent ischemia group revealed no ischemic findings. However, although there was no evidence of any abscess in the permanent ischemia group, intraabdominal adhesions were widespread. The hepatic arteries in all subjects belonging to this group were thrombosed. Histology of the liver revealed highly dilated sinusoids encircling the central vein. The hepatocytes in this region appeared as silhouettes in which nuclear details could not be traced. The nuclei of some hepatocytes which were located more peripherally were seen to contain vacuoles. These hepatocytes, compared with those situated in the central parts of the lobule, were more variable (Fig. 4).

In the patient who underwent intermittent ischemic treatment because of malign mesenchymal tumor metastases in the liver, preoperative ultrasonography revealed several irregularly hypoechoic solid nodular structures in the liver. That the so lesions were reduced in size by ultrasonography has approximately 50% (Table. 2).

**Table 2** Ultrasonographic findings of liver metastases.

| *Date* | *L-1* | *L-2* | *L-3* |
|---|---|---|---|
| 20th May | 55mm | 25mm | 20mm |
| 6thJune | 41 × 34mm | 24 × 20mm | 17 × 15mm |
| 12June | 48 × 30mm | 20 × 18mm | 15 × 13mm |
| 3July | 23 × 19mm | 18 × 17mm | 11 × 12mm |
| 15July | 17 × 13 | 15 × 16mm | 12 × 13mm |
| 12 Aug | 15 × 16mm | 10 × 10mm | No lesion |

During arterial occlusion the patient suffered no discomfort. However, the patient died at home in the second week of September. Since no autopsy was carried out, the exact cause of death is unclear.

### DISCUSSION

The development of arterial collaterals after permanent dearterialization has suggested the need for intermittent dearterialization. The application of transient dearterialization depended on devices that would first block, and than un block the hepatic artery. A variety of devices were prepared to achieve this aim.

One of these devices, the strangulating slings, brought with them several complications such as arterial wall damage and aneurysms[16]. A balloon-catheter placed within the lumen of the hepatic artery, had the disadvantages of hepatic artery thrombosis and required vast doses of heparin for prevention of thrombosis[17,18]. A device that blocks the hepatic artery by exerting external pressure has proved to minimize complications such as aneurysms and thrombosis[40,42].

The device used on subjects in our study was made from a No. 8 Foley catheter. This device produced complete obstruction of the hepatic artery, without any problems.

The vascular occluder (Fig. 2) that was used to obstruct the hepatic artery by external pressure, was very well tolerated by the patient. Although occlu-

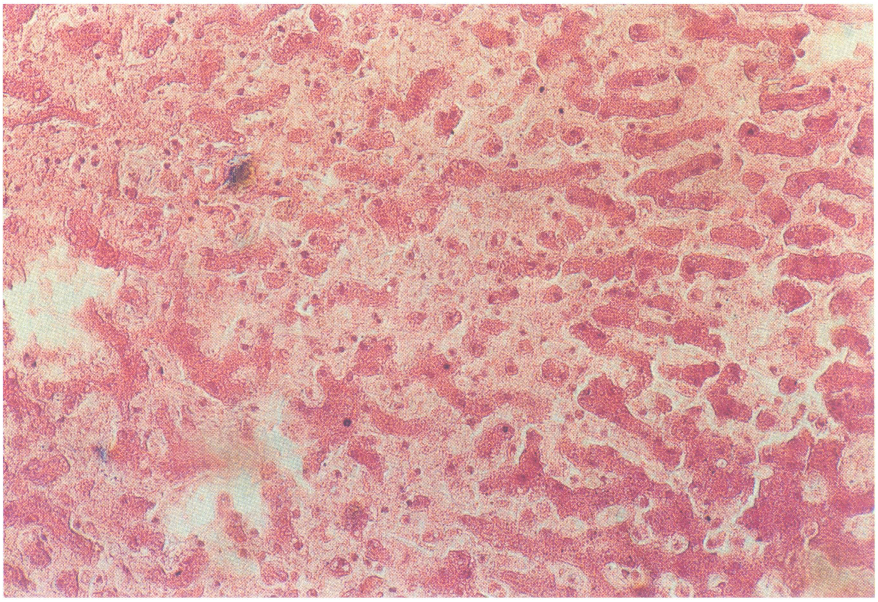

**Figure 4** Seventh dog in permanent ischemia group, with H-E × 10. (See color plate III)

sions were performed in the hospital, it could easily be done by relatives or even the patient himself.

The complete dearteriazation of the liver is an extremely difficult task. It is known that with permanent dearterialization, collaterals develop within a period of 4 days[12]. Collateral vessels developed in 5 subjects in 3-weeks in our permanent ischemia group.

Meanwhile, in the intermittent ischemia group, only one subject among the six which completed 3-weeks period, showed collateral development. No collateral development was observed in the remaining 5 subjects. Fisher's Chi Square Test indicated a marked statistical difference ($p = 0.013$) between the two groups, regarding development of collaterals.

Experimental studies have pointed out that collaternals do not develop with obstruction periods of 2 hours and less, in subjects without tumors[43]. In our study it was shown that collateral development was minimal in subjects without tumors undergoing twice daily, 1 hour occlusion. However, we belive it may be misleading to arrive at a definite conclusion with subjects without a tumor. In cases in which a tumor is present, the vascularity of the tumor is controlled by a variety of angiogenic factors[26]. Angiographic studies carried out in rats both with and without tumors, have indicated that with the obstruction of the common hepatic artery, previously existent arterial connections between the left gastric and hepatic arteries are immediately opened up. These connections are neither dependant on the duration of obstruction, nor is it possible to remove them surgically[27].

The liver biopsies taken from the permanent ischemia group revealed histopathological findings of ischemia; whereas in the intermittent ischemia group there was no change.

Two subject from the permanent ischemia group and one from the intermittent ischemia group died. It was understood that the death of the subjects from the latter group was due to hypovolemia caused by bleeding; however the deaths of the two subjects in the former group (48 hours after the operation) may be due to sepsis from a delay in antibiotic administration. For it is known that there are anaerobic, gram-positive bacilli with spores that reside as saprophytes within the liver of dogs[15].

Although abscess formation is a complication which would be expected to be more frequent in the permanent ischemia group[16,21], it was found in only one subject in the intermittent ischemia group, thus could be due to contamination.

The device which was placed subcutaneously in the patient was very well tolerated, and his treatment proceeded without any complication until his death. The 50% reduction in size of the metastatic lesions can be considered as a partial response. We believe this cannot be attributed solely to the ischemic treatment, nor solely to systemic chemotherapy. The response is most probably due to the combination[7,39].

The results obtained from this study are as follows:

1-Due to swift development of collaterals, ischemia following permanent hepatic dearterialization is temporary.

2-Complete dearterialization of the liver is not possible; however collateral development is minimal after intermittent dearterialization.

3-Intermittent dearterialization is an applicable method and presents a low complication rate.

4-Minimal collateral development is achieved through intermittent occlusions applied twice daily for a period of 60 minutes.

5-Intermittent dearterialization is a method which proves to be well tolerated and has the advantages of being applicable at home by the patient himself.

6-Determination of the effects of ischemic treatment of hepatic tumors is dependant on investigations based on experimental (in tumor bearing animals) and clinical studies and should include comparisons with other treatments.

### REFERENCES

1. Ackerman, N. B: (1972) Experimental studies on the circulatory dynamics of the intrahepatic tumor blood supply. *Cancer*, **29**: 435–439.
2. Ackerman N. B., Lien W. M., Kondi E. S, *et al*: (1969): The blood supply of experimental liver metastases. I. The distribution of hepatic artery and portal vein blood to "small" and "large" tumors. *Surgery*, **66**: 1067–1072.
5. Archer S. G., Gray B. N. (1989): Vascularization of small liver metastases. *Br J Surg*, **76**: 545–548.
7. Bengmark S. (1989) Palliative treatment of hepatic tumours. *Br J Surg*, **76**: 771–773,.
12. Bengmark S., Rosengren K. (1970) Angiographic study of the collateral circulation to the liver after ligation of the hepatic artery in man. *Am J Surg*, **119**: 620–624.
15. Crook J. N., Cohn I. Jr: (1970) Antibiotics and hepatic artery ligaiton in germfree and conventional dogs. *JAMA*, **214**: 343–346.
16. Dahl E. P., Fredlund P. E., Tylén U., Bengmark S. (1981) Transient hepatic dearterialization followed by regional intra-arterial 5-fluorouracil infusion as treatment for liver tumours. *Ann Surg*, **193**: 82–88.
17. El-Domeiri A. A. (1976) A method of intermittent occlusion and chemotherapy infusion of the hepatic artery. *Surg Gynecol Obstet*, **143**: 107–109.
18. El-Domeiri A. A., Mojab K. (1978)Intermittent occlusion of the hepatic artery and infusion chemotherapy for carcinoma of the liver. *Am J Surg*, **135**: 771–775.
20. Gerard A. (1988) Pilot study on the regional treatment of colorectal liver metastases by intermittent arterial ischaemia with degradable starch microspheres and arterial and portal infusion with mitomycin C plus 5-fluorouracil. *Jpn J Cancer Chemother*, **15**: 2627–2632.
21. Jochimsen P. R., Wilbur L. Z., Shirazi S. S., Pearlman N. W. (1978) Iatrogenic liver abscesses: A complication of hepatic artery ligation for tumour. *Arch Surg*, **113**: 141–144.
24. Koehler R. E., Korobkin M., Lewis F. (1975) Arteriographic demonstration of collateral arterial supply to the liver after hepatic artery ligation. *Radiology*, **117**: 49–54.
26. Mack P. (1988) *Repeated Intermittent Hepatic Ischaemia In Liver Cancer Therapy*. An experimental study, Lund.
27. Mack P, Jeppsson B., Rajszys P., *et al*: (1989)Retarding liver cancer growth in the rat by transient repeated hepatic dearterialization. *J Surg Res*, **46**: 123–128.
30. Madding G. F., Kennedy P. A. (1972) Hepatic artery ligation. *Surg Clin North Am*, **52**: 719–728.
31. Mays E. T., Wheeler C. S. (1974) Demonstration of collateral arterial flow after interruption of hepatic arteries in man. *N Engl J Med*, **290**: 993–996.
39. Persson B. G., Jeppsson B., Bengmark S. (1990) DNA-Synthesis after temporary dearterialization of an adenocarcinoma transplanted to the rat liver: A preliminary communication. *Surg Res Comm*, **9**: 163–166.
40. Persson B. G, Nobin A., Ahrén B. *et al.* (1989) Repeated hepatic ischemia as a treatment for carcinoid liver metastases. *World J Surg*; **13**: 307–312.
42. Puntis M. C. A., Persson B. G., Jeppssson B. *et al.* (1987) Free radical production in the ischaemic rat liver. *Surg Res Comm*, **1**: 17–20.
43. Persson B. G., Jeppsson B., Andersson L. *et al.* (1987) The prevention of arterial collateral after repeated temporary blockade of the hepatic artery in pigs. *World J Surg*, **11**: 672–677.
44. Plengvanit U., Chearanai O., Sindhvananda K. *et al.* (1972) Collateral arterial blood supply of the liver after hepatic artery ligaiton, angiographic study of twenty patients. *Ann Surg*,**175**: 105–110.
46. Stein H. J., Oosthuizen M. M. J., Hinder R. A., Lamprechts H. (1991) Oxygen free radicals and glutathione in hepatic ischemia/reperfusion injury. *J Surg Res*, **50**: 398–402.

